# Distinctive sleep complaints and polysomnographic findings in antibody subgroups of autoimmune limbic encephalitis

**DOI:** 10.1007/s10072-024-07652-z

**Published:** 2024-06-24

**Authors:** Cem İsmail Küçükali, Vuslat Yılmaz, Derya Karadeniz, Utku Oğan Akyıldız, Demet İlhan Algın, Ayşegül Şeyma Sarıtaş, Ayşın Kısabay Ak, Aylin Bican Demir, Hikmet Yılmaz, Füsun Mayda Domaç, Ayşe Deniz Elmalı, Ülkü Dübüş Hoş, R. Gökçen Gözübatık-Çelik, Vasfiye Kabeloğlu, Bengisu Bilgin, Deniz Tuncel Berktaş, Bengi Gül Türk, Şakir Delil, Cengiz Dilber, Sedef Terzioğlu Öztürk, S. Naz Yeni, Çiğdem Özkara, Murat Aksu, Erdem Tüzün, Gülçin Benbir Şenel

**Affiliations:** 1 Department of Neuroscience, Aziz Sancar Institute of Experimental Medicine, Istanbul University, Istanbul, Türkiye; 2 Division of Clinical Neurophysiology, Department of Neurology, Istanbul University-Cerrahpasa, Cerrahpasa Faculty of Medicine, Istanbul, Türkiye; 3 Department of Neurology, Adnan Menderes University Medical Faculty, Aydın, Türkiye; 4 Department of Neurology, Eskisehir Osmangazi University Medical Faculty, Eskisehir, Türkiye; 5 Department of Neurology, Celal Bayar University Medical Faculty, Manisa, Türkiye; 6 Department of Neurology, Bursa Uludag University Medical Faculty, Bursa, Türkiye; 7 Department of Neurology, Erenkoy Mental and Nervous Diseases Training and Research Hospital, University of Health Sciences, Istanbul, Türkiye; 8 Department of Neurology, Ministry of Health Basaksehir Cam and Sakura City Hospital, Istanbul, Türkiye; 9 Bakirkoy Prof. Dr. Mazhar Osman Training and Research Hospital for Psychiatric, Neurologic and Neurosurgical Diseases, University of Health Sciences, Istanbul, Türkiye; 10 Department of Pediatrics, Kahramanmaras Sutcu Imam University Medical Faculty, Kahramanmaras, Türkiye; 11 Department of Neurology, Kahramanmaras Sutcu Imam University Medical Faculty, Kahramanmaras, Türkiye; 12 Department of Neurology, Acibadem University Atakent Hospital Medical Faculty, Istanbul, Türkiye

**Keywords:** Autoimmune encephalitis, Sleep disturbances, Polysomnography, Prognosis

## Abstract

**Introduction:**

Sleep disturbances are being increasingly recognized in association with autoimmune encephalitis (AIE). We investigated the prevalence of sleep-related symptoms and polysomnographic features of patients with AIE and the long-term outcomes in these patients in a multi-center, prospective study from Turkey.

**Methods:**

We prospectively evaluated patients with definite AIE in a common database including demographics, AIE-related and sleep-related symptomatology. Maximum and latest modified Rankin scores (mRS) and Liverpool Outcome Score (LOS) were noted.

**Results:**

Of 142 patients, 87 patients (61.3%) fulfilled the criteria for definite AIE (mean age, 46.8+18.8 years; 51.7% women; mean disease duration, 21.0+38.4 months). 78.9% of patients had at least one or more new onset or worsened sleep-related symptomatology: insomnia (55.3%), excessive daytime sleepiness (EDS, 28.0%), sleep apnea (18.7%), REM sleep behavior disorder (RBD, 17.3%), restless legs syndrome (10.7%) and oneiric stupor (9.3%). Sleep efficiency, N3 and REM sleep were decreased and N1 sleep was increased in patients with Ab[+] AIE. LOS points were highest in those with insomnia and sleep apnea, and lowest in those with EDS, RBD and oneiric stupor. RBD and sleep apnea were more common in anti-LG1 Ab[+] group than anti-NMDAR Ab[+] group. Index of periodic leg movements was highest in anti-LG1 Ab[+] group. Patients with EDS and oneiric stupor had more common memory problems. Maximum and latest mRS scores were positively correlated with EDS and oneiric stupor. EDS, RBD and oneiric stupor were negatively correlated with LOS points.

**Conclusion:**

Our study emphasizes the presence and importance of early diagnosis of sleep disturbances in AIE in regard to their deteriorative influences on disease prognosis.

**Supplementary Information:**

The online version contains supplementary material available at 10.1007/s10072-024-07652-z.

## Introduction

Sleep alterations and disorders are being increasingly recognized in association with immune-related neurological disorders. As the emergence of some sleep disturbances are linked to increased inflammation, autoimmune disorders may also affect the central nervous system (CNS), resulting in sleep-related symptoms and disturbances [1]. Autoimmune encephalitis (AIE), which is characterized by a variety of clinical phenotypes in different combinations of encephalopathy, cognitive impairment, epileptic seizures, and movement disorders, is also reported to be associated with sleep alterations and disorders. Sleep-related complaints are usually overlooked in the presence of more prominent AIE symptomatology, and are also challenging due to accompanying mimicking clinical features like cognitive disturbance or seizures. On the other hand, some sleep disorders may have a diagnostic role in some specific AIE, or even play a crucial role in the recognition of AIE, like anti-IgLON5 disease [1]. AIE associated with antibodies against leucine-rich glioma-inactivated 1 (LGI1) or contactin-associated protein 2 (CASPR2), and AIE with Ma2 or IgLON5 autoantibodies are the most widely known and studied subtypes in association with fragmented sleep and sleep disorders such as insomnia, hypersomnia and rapid eye movement (REM) sleep behavior disorder (RBD) [2–4]. However, the overall prevalence of sleep disturbances and their relationship with specific autoantibodies remain largely unknown. In addition, the contributions of sleep alterations and disorders to outcomes in patients with AIE await to be delineated.

There are only few studies in the literature mostly having a retrospective design and with low number of patients, which investigated the spectrum of sleep manifestations in AIE. Blattner et al. [3] reviewed retrospective data of 26 patients with AIE (12 with polysomnography [PSG]), and reported that new sleep-related complaints were common in these patients (present in 73% of the patients), and included snoring, dream enactment behavior, insomnia, and hypersomnia. The authors also reported prominent sleep fragmentation with reduced total sleep time, non-REM sleep stage 3 (N3) and REM sleep. In another study by Erkent et al. [5], 17 patients with AIE who had sleep complaints with acute or subacute onset and referred to sleep laboratory for PSG recordings were retrospectively investigated. The authors showed that in addition to the alterations in sleep structure, a broad spectrum of sleep disorders was present in AIE associated with different antibodies, and nearly half of the patients had acute or subacute-onset sleep related breathing disorders. Long-term follow-up data in these patients with AIE and the prognostic role of sleep alterations and/or disorders are not studied in detail. Also, distinctive features of sleep disorders in different antibody subgroups of autoimmune limbic encephalitis have not been sufficiently investigated.

In this study, we aimed to investigate the prevalence of sleep-related symptoms and polysomnographic characteristics in patients with AIE in a multi-center, prospective study in Turkey. We also aimed to investigate the long-term outcomes of these patients in regard to contributions of sleep alterations and disorders in AIE associated with different antibodies.

## Materials and methods

### Participants and clinical evaluation

Our study was conducted between the years 2022 and 2023, and the patients diagnosed as having definite AIE were prospectively enrolled. Clinical examinations, diagnostic work-up and the follow-up evaluations were made by the primary physicians of the patients in each center, and then collected by the primary investigator (G.B.S.). All of the patients included were admitted to the Emergency Room, and were being hospitalized in Neurology inpatient clinics. None of these patients were admitted to intensive care unit (ICU) before sleep analysis. On the other side, during follow-up periods, four patients were consulted with ICU due to clinical deterioration, and unfortunately died of disease-related complications. The study was approved by the Local Ethical Committee of Istanbul University-Cerrahpasa, and all participants or their relatives gave their written informed consents for the participation into the study.

The diagnosis of AIE was made based on the diagnostic criteria for definite autoimmune limbic encephalitis defined by Graus et al. [6]. NMDAR-antibody positive AIE patients also fulfilled the criteria for definite NMDAR encephalitis. All patients were examined by a neurologist for altered mental status, deficits in working memory, psychiatric problems or focal CNS findings. In addition to detailed clinical anamnesis, existing medical records were reviewed. A common database was formed including demographic data, detailed questioning of AIE-related symptoms, and sleep-related symptomatology. Demographic and clinical data included sex, age on admission, disease duration (starting from disease onset till the latest follow-up point), presenting symptom, and the presence of encephalopathy (altered mental status and personality change), memory problems, psychiatric and psychotic features, catatonia, mutism, epileptic seizures, dystonia or other movement disorders, autonomic disturbances (like excessive sweating or bladder dysfunction) and accompanying endocrine problems (such as thyroiditis and diabetes mellitus type 1). All patients had cranial magnetic resonance imaging (MRI), electroencephalography (EEG), and cerebrospinal fluid (CSF) analysis. Patients were not involved if alternative causes of encephalitis could not be reasonably excluded. Follow-up data included all treatments given during disease duration by the primary physician, the maximum modified Rankin scores(mRS) during the disease course, latest mRS during hospital discharge and Liverpool Outcome Score(LOS) for assessing treatment response of adults at Discharge from the hospital (https://www.liverpool.ac.uk/media/livacuk/infectionandglobalhealth/braininfections/DischargeLOS_ADULTS.pdf) [7]. The final LOS is the lowest number scored for any single question; 5 points for full recovery & normal neurological examination, 4 points for minor sequelae with mild effects on function or personality change or on medication, 3 points for moderate sequelae mildly affecting function, probably compatible with independent living, 2 points for severe sequelae, impairing function sufficient to make patient dependent, and 1 point for death. Sleep-related complaints were queried to all patients and/or relatives, and included premorbid sleep diagnosis, new onset or worsened symptoms like insomnia, excessive daytime sleepiness, dream enactment behavior suggestive of RBD, snoring, witnessed apnea or other symptoms suggestive of sleep apnea, and restless legs syndrome (RLS). Upon the questioning of sleep complaints and polysomnography, the diagnoses of sleep disorders were made on the basis of the International Classification of Sleep Disorders 3^rd^ edition [8].

### Antibody assays

Autoantibody testing was made for the known autoimmune encephalitis-related antibodies, using the commercial Euroimmun kits (Luebeck, Germany). These included autoantibodies against N-Methyl-D-Aspartate receptor (NMDAR), α-amino-3-hydroxy-5-methyl-4-isoxazolepropionic acid receptor (AMPAR), leucine rich glioma inactivated 1 (LGI1), contactin associated protein 2 (CASPR2), Gamma-Aminobutyric Acid-B receptor (GABABR), glutamic acid decarboxylase (GAD), DPPX and IgLON5. In addition, paraneoplastic antibodies, including amphiphysin, Ma2, CV2, Hu, Yo (Purkinje cell antibody 1, PCA-1) and Ri (anti-neuronal nuclear antibody 2, ANNA-2) were tested. Antibody assays were conducted in all serum (*n*=87) and available CSF samples (*n*=36) of patients. Serum antibody results of patients whose CSF samples were not available for antibody investigation were confirmed by cell-based assays (utilizing live HEK cells) and tissue-based assays (utilizing indirect immunohistochemistry on frozen rat brain sections), as reported previously [9–11].

### Polysomnography

Polysomnography was performed during the hospitalization in patients with AIE if they had a newly-emerging active sleep complaints with a clear clinical indication. Factors that may influence sleep quality and polysomnographic parameters, including acute infections, unstable vital signs, or use of drugs affecting our analysis, like opioids, were strictly interrogated and excluded. The evaluation of PSG and sleep parameters was made by an experienced clinical neurophysiologist and sleep expert in each center (certified by the Turkish Sleep Medicine Society) based on the latest version of the American Academy of Sleep Medicine (AASM) Manual [12]. In PSG recordings, total recording time (TRT), total sleep time (TST), sleep efficiency (SE), sleep latency (SL), REM sleep latency (REML), wakefulness after sleep onset (WASO), the durations and percentages of sleep stages (N1, N2, N3 and REM), apnea-hypopnea index (AHI) for the obstructive and central events, mean and minimum oxygen saturation, index of periodic limb movements in sleep, and the presence of REM sleep without atonia (RSWA). For the comparison of PSG data, a sex- and age-matched healthy control group (*n*=26) was randomly selected from the sleep laboratory of the principal investigator.

### Statistical analysis

All medical records of the participants were shared by the primary investigator, who reviewed the data in terms of consistency and diagnostic accuracy. Data was analyzed by using the Statistical Package for the Social Sciences version 20.0 (IBM® SPSS 20.0). Data was given as numbers and percentages for the categorical variables, as mean + standard deviation for the continuous variables with normal distribution, and as median and interquartile range (IQR) for the continuous variables without normal distribution. Comparisons of categorical and continuous parameters were made by using the Fisher’s exact test and the Mann–Whitney U test, correspondingly. For the multiple comparisons, ANalysis Of VAriance (ANOVA) test was performed with post-hoc Bonferroni (for the comparisons of groups with unequal numbers) or Tamhane’s T2 (for the parameters without normal distribution) tests. Pearson or Spearman correlation coefficients were used in the correlation analyses of the parameters with or without normal distribution, respectively. Age, sex and the type of antibody was corrected in correlation analyses, by entering the confounders one-by-one in stepwise fitting model. A *p* value of equal to or below 0.05 was regarded as statistically significant.

## Results

### Analysis of all participants

Of 142 patients investigated with a suspicion of AIE, 87 patients (61.3%) fulfilled the criteria for definite autoimmune limbic encephalitis. Mean age of patients with AIE was 46.8+18.8 years with a mean disease duration (from first symptom to latest follow-up) of 21.0+38.4 months. 51.7% of the patients (*n*=45) were women. The frequency of symptoms related to AIE was as follows: altered mental status or personality change (encephalopathy) in 69.0%, memory problems in 62.1%, psychiatric symptoms in 67.8%, catatonia/mutism in 6.9%, epileptic seizures in 54.0%, dystonia or other movement disorders in 20.7%, autonomic symptoms in 26.4%, and endocrine disturbances in 19.5%. The most common sleep-related complaint in whole group was insomnia (55.3%), followed by excessive daytime sleepiness (28.0%), sleep apnea (18.7%), REM sleep behavior disorder (17.3%), restless legs syndrome (10.7%) and oneiric stupor (9.3%). Overall, 78.9% of all patients with AIE had at least one or more new onset or worsened sleep-related symptomatology.

Forty-three patients (49.4%) had anti-NMDAR Ab; 22 patients (25.3%) had anti-LG1/CASPR2 Ab (13 patients with anti-LG1 Ab, 5 patients with anti-CASPR2 Ab and 4 patients with both); 4 patients (4.6%) had anti-GABA-BR Ab; 3 patients (3.4%) had anti-GAD Ab; 2 patients (2.3%) had anti-IgLON5 Ab; one patient (1.1%) had both anti-Hu and CV2 Abs; and one patient (1.1%) had anti-Ri Ab. Eleven patients (12.6%) had the diagnosis of autoantibody-negative definite autoimmune limbic encephalitis. The average maximum mRS score during the disease duration was 3.1+1.4 points in whole group, and the average final mRS score of the patients was 2.0+1.8 points. About 75.4% of the patients showed total or almost total improvement after the treatment (LOS point, 4 and 5), while four patients (5.2%) died (LOS point, 1). The time from PSG to LOS and final mRS was calculated as 2.2+3.1 months.

### Analysis of antibody-positive and antibody-negative patients with AIE

**Fig. 1:**
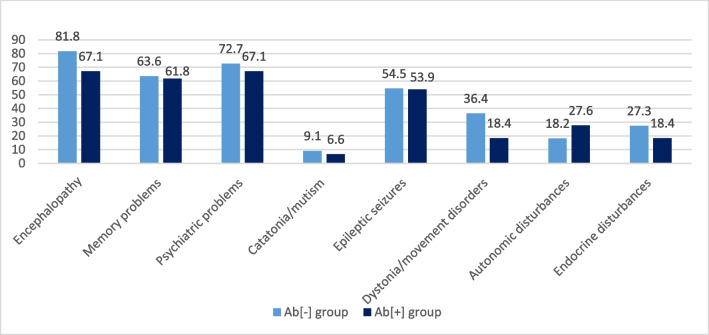
The frequency (%) of AIE-related symptoms between Ab[-] and Ab[+] groups. *p values: encephalopathy, *p*=0.270; memory problems, *p*=0.593; psychiatric problems, *p*=0.502; catatonia/mutism, *p*=0.567, epileptic seizures, *p*=0.614; dystonia or other movement disorders, *p*=0.163; autonomic disturbances, *p*=0.400; endocrine disturbances, *p*=0.366

**Fig. 2:**
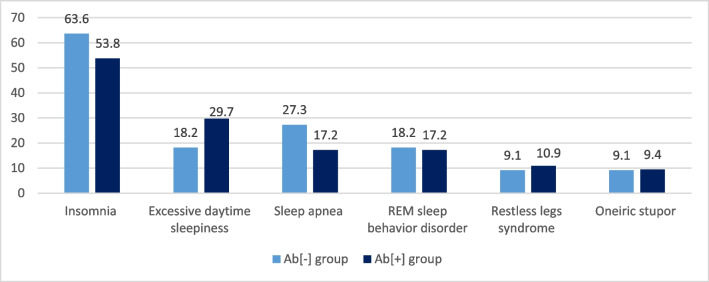
The frequency (%) of sleep-related symptoms between Ab[-] and Ab[+] groups. *p values: insomnia, *p*=0.395; excessive daytime sleepiness, *p*=0.350; sleep apnea, *p*=0.335; REM sleep behavior disorder, *p*=0.611; restless legs syndrome, *p*=0.667; oneiric stupor, *p*=0.728

Patients were grouped into autoantibody-negative (Ab[-], *n*=11) and autoantibody-positive definite AIE (Ab[+], *n*=76) groups. Mean age (*p*=0.942), age at disease onset (*p*=0.839), disease duration (*p*=0.181) and sex (*p*=0.550) were indifferent between two groups. The frequency of AIE-related (Fig. 1) and sleep-related (Fig. 2) symptoms showed no significant differences between Ab[-] and Ab[+] groups.

In PSG parameters (Table 1), sleep efficiency was significantly lower in patients with Ab[+] AIE in compared to healthy controls. The percentage of N1 sleep was significantly increased in patients with Ab[+] AIE in compared to those with Ab[-] AIE and controls. In patients with Ab[-] AIE, on the other side, REM sleep latency was significantly increased in compared to those with Ab[+] AIE and controls. The percentage of REM sleep was significantly lower in patients with Ab[+] and Ab[-] AIE than those in controls. In addition, patients with Ab[+] AIE revealed a significantly higher AHI in compared to controls.

**Table 1:** Comparisons of polysomnographic parameters between Ab[-] and Ab[+] groups, and in compared to healthy control subjects

PSG parameters	Ab[-] group (*n*=11)	Ab[+] group (*n*=76)	*p* values*	Control group (*n*=26)	*p* values^#^
TRT (min)	496.8+84.2	522.0+238.2	0.999	466.8+27.2	0.476
TST (min)	340.4+118.4	374.4+157.9	0.764	393.0+63.6	0.570
SE (%)	73.2+20.8^a,b^	69.0+18.8^a^	0.595	81.8+12.8^b^	**0.030**
SL (min)	34.2+31.9	52.4+103.8	0.729	17.4+16.7	0.227
REML (min)	254.2+77.4^a^	143.6+84.7^b^	**0.004**	112.8+77.0^b^	**0.001**
WASO (min)	137.0+105.8	164.2+187.5	0.999	85.8+64.0	0.129
N1 sleep (%)	8.2+10.2^a^	12.9+11.4^b^	**0.043**	8.7+3.6^a^	**0.037**
N2 sleep (%)	37.3+15.2^a^	40.3+17.2^a,b^	0.835	47.4+8.2^b^	**0.036**
N3 sleep (%)	34.6+26.6	18.4+13.8	0.174	23.4+5.2	0.176
R sleep (%)	9.7+6.2^a^	11.6+6.9^a^	0.672	16.8+6.2^b^	**0.008**
AHI (per hour)	6.0+11.6^a,b^	12.6+15.0^a^	0.061	4.3+3.6^b^	**0.031**
Mean O_2_ saturation (%)	94.8+2.4	95.0+0.2	0.872	95.7+1.4	0.374
Minimum O_2_ saturation (%)	85.5+5.5	85.4+8.8	0.642	88.2+4.1	0.478
PLMSI (per hour)	5.0+4.2	17.1+19.9	0.529	9.0+11.2	0.253

bold for statistical significance.

*TRT* total recording time, *TST* total sleep time, *SE* sleep efficiency, *SL* sleep latency, *REML* REM sleep latency, *WASO* wakefulness after sleep onset, *N1* NREM sleep stage 1, *N2* NREM sleep stage 2, *N3* NREM sleep stage 3, *R* NREM sleep stage REM, *AHI* apnea-hypopnea index, *PLMSI* index of periodic leg movements in sleep.

*p values between Ab[-] and Ab[+] groups.

^**#**^*p* values between Ab[-], Ab[+] and control groups - ^a-b^ not statistically different for the parameters with the same letter for each test.

Maximum (*p*=0.118) and latest mRS scores (*p*=0.417) did not show statistically significant differences between Ab[-] and Ab[+] groups. Benefit from treatment was also similar between two groups (*p*=0.215), though all of four deceased patients (LOS point, 1) were in the Ab[+] group. On the other hand, in patients with Ab[+] AIE, LOS points were highest in those with insomnia (*p*=0.020) and sleep apnea (*p*=0.032), and they were lowest in those with excessive daytime sleepiness (*p*=0.033), RBD and oneiric stupor (*p*=0.001).

### Analysis of patients with AIE in different antibody subgroups

**Fig. 3:**
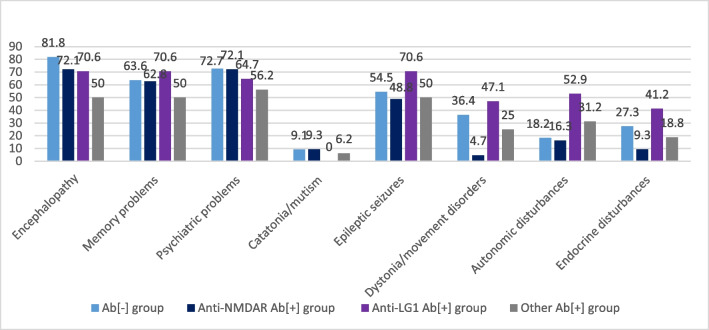
The frequency (%) of AIE-related symptoms among AIE patients with different Ab subgroups. *p values: encephalopathy, *p*=0.298; memory problems, *p*=0.685; psychiatric problems, *p*=0.685; catatonia/mutism, *p*=0.640, epileptic seizures, *p*=0.497; dystonia or other movement disorders, *p*=0.001; autonomic disturbances, *p*=0.028; endocrine disturbances, *p*=0.038

Patients were grouped into four as antibody-negative (Ab[-] group, *n*=11), anti-NMDAR Ab[+] group (*n*=43), anti-LG1 Ab[+] group (*n*=17), and other Ab[+] group (*n*=16). Patients with both LGI1 and/or CASPR2-Ab were grouped together as the LGI1-Ab group. Mean age of the patients was observed to be the lowest in anti-NMDAR Ab[+] group (41.5+18.2 years), and to be highest in anti-LG1 Ab[+] group (53.4+17.2 years; *p*=0.078). Mean age at disease onset (*p*=0.217), disease duration (*p*=0.353) and sex (*p*=0.653) were also indifferent among four groups. Of AIE-related symptoms, dystonia or other movement disorders (*p*=0.001), autonomic disturbances (*p*=0.028), and endocrine disturbances (*p*=0.038) showed significant differences among four groups. Post-hoc analysis showed that these differences resulted from two subgroups; anti-NMDAR and anti-LG1 Ab[+] groups. Anti-NMDAR Ab[+] group had the lowest prevalence for dystonia or other movement disorders(4.7%), autonomic(16.3%) and endocrine disturbances (9.3%), while anti-LG1 Ab[+] group had the highest prevalence for dystonia or other movement disorders (47.1%), autonomic (52.9%) and endocrine disturbances (41.2%) (*p*=0.024, *p*=0.022 and *p*=0.030; respectively). Ab[-] group and other Ab[+] group were placed in between these two groups, and they did not show significant differences as compared to any other group (Fig. 3).

**Fig. 4:**
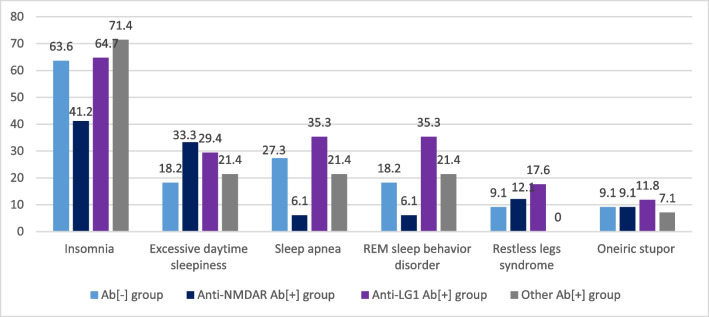
The frequency (%) of sleep-related symptoms among AIE patients with different Ab subgroups. *p values: insomnia, *p*=0.167; excessive daytime sleepiness, *p*=0.739; sleep apnea, *p*=0.067; REM sleep behavior disorder, *p*=0.074; restless legs syndrome, *p*=0.464; oneiric stupor, *p*=0.979

Sleep-related complaints showed that insomnia (*p*=0.167), excessive daytime sleepiness (*p*=0.739), restless legs syndrome (*p*=0.464) and oneiric stupor (*p*=0.979) were similar among four groups. On the other hand, sleep apnea (*p*=0.067) and REM sleep behavior disorder (*p*=0.074) showed some differences close to the statistically significant level. Post-hoc analysis of sleep apnea and REM sleep behavior disorder showed that their prevalence was lowest in anti-NMDAR Ab[+] group, and highest in anti-LG1 Ab[+] group, but not significantly (*p*=0.061 and *p*=0.073, respectively) (Fig. 4). On the other hand, REM sleep behavior disorder (6.1% versus 28.6%, *p*=0.031; consecutively) and sleep apnea (6.1% versus 30.8%, *p*=0.013; consecutively) were significantly more common in anti-LG1 Ab[+] group than those in anti-NMDAR Ab[+] group.

Comparisons of PSG parameters among four groups are given in Table 2. Except for REM sleep latency (*p*=0.025) and the index of periodic leg movements (*p*=0.034), PSG parameters were also not significantly different among groups. Post-hoc analysis showed that REM sleep latency was highest in Ab[-] group, and lowest in anti-LG1 Ab[+] group (*p*=0.015). Mean index of periodic leg movements was lowest in Ab[-] group and highest in anti-LG1 Ab[+] group (*p*=0.022).

**Table 2:** Comparisons of polysomnographic parameters among four groups

PSG parameters	Ab[-] group (*n*=11)	Anti-NMDAR Ab[+] group (*n*=43)	Anti-LG1 Ab[+] group (*n*=17)	Other Ab[+] group (*n*=16)	*p* values
TRT (min)	496.8+84.2	391.7+175.5	625.2+217.7	529.7+304.8	0.423
TST (min)	340.4+118.4	361.6+103.6	413.6+174.1	308.6+197.0	0.469
SE (%)	73.2+20.8	73.4+15.0	68.4+21.5	63.4+20.2	0.780
SL (min)	34.2+31.9	68.6+111.6	14.7+10.6	109.4+177.3	0.276
REML (min)	254.2+77.4	164.8+109.0	115.4+65.0	160.4+73.0	**0.023**
WASO (min)	137.0+105.8	102.1+50.0	198.7+222.6	175.4+235.8	0.920
N1 sleep (%)	8.2+10.2	7.8+4.2	17.2+15.6	11.7+1.9	0.065
N2 sleep (%)	37.3+15.2	37.1+8.2	43.4+19.8	38.5+23.4	0.895
N3 sleep (%)	34.6+26.6	25.0+11.5	12.6+12.4	19.2+17.1	0.190
R sleep (%)	9.7+6.2	11.0+5.8	11.4+9.2	13.0+4.6	0.812
AHI (per hour)	6.0+11.6	8.4+10.4	11.9+11.2	20.6+26.0	0.153
Mean O_2_ saturation (%)	94.8+2.4	95.8+1.9	95.0+2.4	93.6+2.4	0.416
Minimum O_2_ saturation (%)	85.5+5.5	87.8+4.4	84.2+11.4	84.2+8.4	0.879
PLMSI (per hour)	5.0+4.2	8.7+9.6	27.4+21.9	0.8+1.3	**0.034**

bold for statistical significance.

*TRT* total recording time, *TST* total sleep time, *SE* sleep efficiency, *SL* sleep latency, *REML* REM sleep latency, *WASO* wakefulness after sleep onset, *N1* NREM sleep stage 1, *N2* NREM sleep stage 2, *N3* NREM sleep stage 3, *R* NREM sleep stage REM, *AHI* apnea-hypopnea index, *PLMSI* index of periodic leg movements in sleep.

Maximum (*p*=0.850) and latest mRS scores (*p*=0.819), and the ratio of benefits from treatment (*p*=0.766) were similar among groups. LOS points were highest in anti-NMDAR Ab[+] group, and lowest in other Ab[+] group (*p*=0.087).

Analyses of patients with AIE associated with anti-LG1 Ab[+] and anti-CASPR2 Ab[+] are given in Supplement data 1.

### Correlation analyses

Correlation analyses between AIE-related and sleep-related symptoms showed patients with excessive daytime sleepiness (*r*=0.420, *p*<0.001) and oneiric stupor (*r*=0.248, *p*=0.032) had more common memory problems. Patients with excessive daytime sleepiness also more commonly displayed encephalopathy (*r*=0.323, *p*=0.005) and catatonia/mutism (*r*=0.254, *p*=0.028). The PSG parameters, however, failed to show significant correlations with AIE-related symptoms. From prognostic point of view, maximum mRS scores were positively correlated with excessive daytime sleepiness (*r*=0.280, *p*=0.031) and oneiric stupor (*r*=0.320, *p*=0.013); and the latest mRS scores were positively correlated with excessive daytime sleepiness(*r*=0.375, *p*=0.003). In parallel to these findings, excessive daytime sleepiness(*r*=-0.224, *p*=0.044), RBD (*r*=-0.247, *p*=0.038), and oneiric stupor (*r*=-0.235, *p*=0.052) showed significant negative correlations with LOS points.

## Discussion

Our study analyzed the clinical features and sleep characteristics in 87 patients with definite autoimmune limbic encephalitis, with a mean disease duration of almost two years. We demonstrated that sleep-related disturbances were very common in patients with AIE, with a prevalence of 78.9%; the most common one being insomnia (55.3%), followed by excessive daytime sleepiness (28.0%), sleep apnea (18.7%), REM sleep behavior disorder (17.3%), restless legs syndrome (10.7%) and oneiric stupor (9.3%). It was previously studied in small cohorts and reported that sleep disturbances were common in patients with AIE. In a study by Blattner et al. [3], it was reported that 73% of patients with AIE had new or worsened sleep-related symptoms in a cohort of 26 patients with AIE associated with different subgroups of autoantibodies. The authors stated that gasping or snoring was the most common sleep-related complaint, followed by dream enactment behaviors, insomnia, hypersomnia, other parasomnias, and confusional dream-wake states. In a very recent study from one center in Turkey by Erkent et al. [5], 17 patients with AIE associated with different subgroups of antibodies were prospectively investigated for the new onset or worsened sleep disturbances. The authors found that all of their patients had at least one sleep-related complaint and/or disorder, excessive daytime sleepiness and abnormal movements during sleep being the most common ones; followed by snoring, insomnia, and parasomnias. In this regard, our multicenter study with 87 participants supports previous findings and demonstrates that a wide variety of sleep disturbances commonly accompanies AIE, emphasizing the need for a specific questioning of sleep-related symptoms in these patients, as they may easily be unrecognized next to catastrophic AIE-related clinical features, or due to overlapping symptomatology.

We also investigated the prognostic value of sleep-related symptoms in patients with AIE, for the first time in the literature. We observed that maximum mRS scores were positively correlated with the presence of excessive daytime sleepiness and oneiric stupor. The latest mRS scores after a mean of two years of disease duration were also positively correlated with excessive daytime sleepiness. In parallel to these findings, excessive daytime sleepiness, RBD and oneiric stupor showed significant negative correlations with treatment outcome as measured by the LOS points. These data emphasize the importance of early diagnosis of sleep disturbances in patients with AIE, and warrant the need for their treatment. The effects of early recognition and initiation of treatments for associated sleep disturbances on short and long-term prognosis of AIE, however, need to be explored.

In two groups of antibody-positive and antibody-negative patients with AIE, we did not find significant differences in terms of demographic parameters, prevalence of AIE-related or sleep-related symptoms. The analysis of patients with AIE in different antibody subgroups, however, revealed that patients with AIE associated with anti-NMDAR antibodies had the lowest prevalence of dystonia or other movement disorders, autonomic disturbances and endocrine disturbances, while patients with AIE associated with anti-LG1(+CASPR2) antibodies had the highest prevalence. Sleep apnea and RBD was also lowest in patients with AIE associated with anti-NMDAR antibodies, and highest in patients with AIE associated with anti-LG1 (+CASPR2) antibodies, but not significantly. Furthermore, the comparison of the patients with AIE associated with anti-LG1 and anti-CASPR2 antibodies revealed that none of those with anti-CASPR2 antibodies had dystonia or other movement disorders, while it was significantly common in patients with AIE associated with anti-LG1 antibodies. RBD was not reported in patients with AIE associated with anti-CASPR2 antibodies, while it was significantly more common in patients with AIE having both anti-LG1 and anti-CASPR2 antibodies.

In the literature, insomnia was reported to be more common in AIE associated with anti-NMDAR Ab, anti-CASPR2 Ab, and less frequently with anti-LGI1 Ab [13, 14]. Dream enactment behaviors and RBD were reported in AIE associated with many different autoantibodies, including anti-LGI1, anti-CASPR2, anti-IgLON5, and anti-Ma2 [3, 14]. Bizarre, irregular movements and periodic limb movements during wakefulness and/or sleep, however, were reported to be related to AIE associated with anti-IgLON5 or anti-DPPX antibodies [13, 15–17]. Sleep apnea was reported to be typical to AIE associated with anti-IgLON5 antibodies [13, 16], though snoring, gasping or witnessed apnea were also reported in other types of AIE, such as in association with anti-AMPA antibodies [3], anti-DPPX antibodies [17], or in association with anti-LG1 antibodies, as demonstrated in our study. Similarly, excessive daytime sleepiness due to hypocretin deficiency was very closely linked to AIE associated with anti-Ma2 antibodies, while excessive daytime sleepiness, as an isolated symptom, may occur in association with other subtypes of antibodies in patients with AIE. Taken together with the prior results in the literature, our findings support that some subgroups of autoantibodies are associated with increased risk of specific sleep-related disturbances.

Although the pathophysiology of sleep disturbances in AIE are still speculative [18], involvement of a wide variety of cerebral localizations in AIE may explain the broad spectrum of sleep disorders in these patients. In a recent study by Liu et al. [19], positron emission tomography showed a widespread metabolic dysfunction in regions of interest, which correlated well with the sleep quality in these patients. Such as, lesions in the reticular activating system may be responsible for insomnia and excessive daytime sleepiness, while lesions affecting hypothalamus, thalamus and limbic system may result in RBD, or medullary lesions affecting chemoreceptors and peripheral baroreceptors may predispose sleep apnea. In our cohort, correlation analyses between AIE-related and sleep-related symptoms showed that patients with excessive daytime sleepiness and oneiric stupor had more memory problems. Patients with excessive daytime sleepiness also more commonly displayed encephalopathy and catatonia/mutism. To the authors’ knowledge, this correlation between AIE-related clinical features and sleep disturbances was not investigated before. While further studies should be performed to delineate the underlying pathophysiology to better understand these correlations, excessive daytime sleepiness may suggest the involvement of medial limbic circuits, and the presence of oneiric stupor may suggest the involvement of retrosplenial cortex of limbic circuits in particular [20], which may explain memory problems and encephalopathy being more prominent in these patients.

The evaluation of objective sleep measures by polysomnography showed that sleep efficiency was significantly lower, and the duration of N1 sleep was significantly higher in antibody-positive patients with AIE in compared to healthy controls; indicating fragmented sleep and decreased sleep quality in these patients. Interestingly, REM sleep latency was significantly increased in antibody-negative patients with AIE. The duration of N3 and REM sleep was also decreased in all patients with AIE, but the only significance was observed in REM sleep. It was also observed that shortening of REM sleep latency was significant and prominent in patients with AIE associated with anti-LG1 (+CASPR2) antibodies. Of limited number of PSG studies in small cohorts of patients with AIE, altered sleep architecture characterized by decreased total sleep time, sleep efficiency and duration of N3 and REM sleep stages, and increased duration of N1 sleep with increased sleep fragmentation were almost uniformly reported in patients with AIE associated with different subgroups of antibodies [3, 5, 19]. These data show that the sleep architecture is prominently affected in patients with AIE associated with different subgroups of antibodies. In addition, disruptions in sleep structure with prominently decreased sleep efficiency and durations of N3 and REM sleep in patients with AIE were suggested to contribute to memory impairments and attention deficits, and for this reason, worsen the functional outcome in these patients [21, 22]. In our study, we failed to show a significant correlation between PSG parameters and AIE-related memory problems.

We also observed a significantly higher AHI was observed in antibody-positive patients with AIE in comparison to controls. Intriguingly, mean index of periodic leg movements was significantly increased in patients with AIE associated with anti-LG1 (+CASPR2) antibodies. Data regarding the indices of apnea-hypopneas and periodic leg movements in sleep are very scarce in the literature. Lin et al. [23] prospectively investigated 27 patients with encephalitis associated with anti-LGI1 antibodies and 7 patients with anti-CASPR2 disease, and reported that RBD and periodic limb movements in sleep were more frequent in patients with AIE associated with anti-LGI1 antibodies. This finding requires further investigation about whether these antibodies have a unique or increased affinity to circuits responsible from the generation of periodic leg movements in sleep, particularly the central pattern generators.

Our study revealed some new and interesting data on sleep disorders in autoimmune encephalitis characterized by a variety of clinical phenotypes and auto-antibodies. Our study also has some potentials to provide information on the disease pathophysiology, and hints for a better understanding of the mechanisms of some sleep disorders. On the other side, the small sample size of subgroups is a major limitation of our study which makes it difficult to make reliable conclusions. Studies with larger cohorts are warranted to support our findings.

## Supplementary information

ESM 1 (DOCX 53 kb)
