# Supplementary material for: Distinctive sleep complaints and polysomnographic findings in antibody subgroups of autoimmune limbic encephalitis
Source: Neurol Sci. 2024 Jun 24;45(11):5429–39. doi: 10.1007/s10072-024-07652-z (PMC11470856; doi:10.1007/s10072-024-07652-z)
Supplement: Supplementary file 1 — (DOCX 53 kb) [file 10072_2024_7652_MOESM1_ESM.docx]

***Supplement Data 1. Analysis of Patients with AIE Associated with Anti-LG1 Ab and Anti-CASPR2 Ab***

Of 13 patients with anti-LG1 Ab[+], 53.8% were females, and of five patients with anti-CASPR2 Ab[+] 80% were males; four patients had both anti-LG1 and anti-CASPR2 antibodies, and male to female ratio was 1:1 (p=0.425). Mean age on admission (p=0.079) and age at disease onset (p=0.231) was not statistically different among the groups. On the other hand, mean disease duration was longest in patients with anti-CASPR2 Ab[+] (23.2+26.2 months), which was 14.6+9.4 months in patients with anti-LG1 Ab[+], and 3.0+1.2 months in patients having both antibodies (p=0.029).

Dystonia or other movement disorders were not present in any of the patients with anti-CASPR2 Ab[+], and it was most commonly observed in patients with anti-LG1 Ab[+] (p=0.041). Memory problems were also most commonly observed in patients with anti-LG1 Ab[+], and least commonly in patients with anti-CASPR2 Ab[+] (p=0.082). Other AIE-related symptoms were similar among three groups (Fig.1). Comparisons of sleep-related complaints revealed that REM sleep behavior disorder was not reported in patients with anti-CASPR2 Ab[+], and it was most commonly detected in patients having both antibodies(p=0.049). Insomnia was present in all four patients having both antibodies, and least common in patients with anti-CASPR2 Ab[+] (p=0.093). Excessive daytime sleepiness was present only in patients with anti-LG1 Ab[+] (p=0.133, Fig.2).

PSG parameters among these three groups are given in Table 1. Mean index of periodic leg movements in sleep was prominently lower in patients with anti-CASPR2 Ab[+] in compared to the other two groups, but not significantly. Sleep structure and other PSG parameters were similar among three groups.

**Table 1.** Comparisons of polysomnographic parameters between AIE patients with anti-LG1 and/or anti-CASPR2 antibodies.

| PSG parameters | Patients with Anti-LG1 Ab[+] AIE  (n=13) | Patients with Anti-CASPR2 Ab[+] AIE  (n=5) | Patients with Anti-LG1 and  Anti-CASPR2 Ab[+] AIE  (n=4) | p values |
| --- | --- | --- | --- | --- |
| TRT (min) | 589.8+244.4 | 441.7+178.0 | 687.2+174.5 | 0.535 |
| TST (min) | 373.1+139.9 | 325.6+114.1 | 484.5+226.6 | 0.393 |
| SE (%) | 68.2+25.0 | 82.2+18.3 | 68.8+17.2 | 0.823 |
| SL (min) | 14.8+13.0 | 28.8+114.0 | 14.6+5.9 | 0.308 |
| REML (min) | 109.9+54.9 | 194.8+99.5 | 126.3+95.6 | 0.470 |
| WASO (min) | 222.6+277.6 | 138.8+137.4 | 157.0+89.0 | 0.720 |
| N1 sleep (%) | 11.2+7.6 | 8.8+6.6 | 27.6+21.6 | 0.174 |
| N2 sleep (%) | 44.2+23.3 | 36.4+14.2 | 42.1+14.7 | 0.720 |
| N3 sleep (%) | 12.8+14.2 | 27.5+19.6 | 12.2+9.6 | 0.774 |
| R sleep (%) | 11.4+8.8 | 10.9+5.6 | 11.5+11.7 | 0.872 |
| AHI (per hour) | 9.0+5.1 | 10.6+16.6 | 17.1+17.5 | 0.824 |
| Mean O_2_ saturation (%) | 94.0+2.4 | 94.9+2.4 | 97.0+0.8 | 0.116 |
| Minimum O_2_ saturation (%) | 85.2+7.0 | 86.0+6.0 | 83.0+16.7 | 0.625 |
| PLMSI (per hour) | 27.8+21.1 | 2.3+5.6 | 26.7+28.0 | 0.299 |

*TRT: total recording time; TST: total sleep time; SE: sleep efficiency; SL: sleep latency; REML: REM sleep latency; WASO: wakefulness after sleep onset; N1: NREM sleep stage 1; N2: NREM sleep stage 2; N3: NREM sleep stage 3; R: NREM sleep stage REM; AHI: apnea-hypopnea index; PLMSI: index of periodic leg movements in sleep.

**Figure 1.** The frequency (%) of AIE-related symptoms between AIE patients with anti-LG1 and/or anti-CASPR2 antibodies.

*p values: encephalopathy, p=0.448; memory problems, p=0.082; psychiatric problems, p=0.769; catatonia/mutism, p=0.999; epileptic seizures, p=0.543; dystonia or other movement disorders, p=0.041; autonomic disturbances, p=0.528; endocrine disturbances, p=0.512.

**Figure 2.** The frequency (%) of sleep-related symptoms between AIE patients with anti-LG1 and/or anti-CASPR2 antibodies.

*p values: insomnia, p=0.093; excessive daytime sleepiness, p=0.133; sleep apnea, p=0.718; REM sleep behavior disorder, p=0.049; restless legs syndrome, p=0.590; oneiric stupor, p=0.453.
